# DMM Outstanding Paper Prize 2021 winner: Daniel Bronder

**DOI:** 10.1242/dmm.049607

**Published:** 2022-05-03

**Authors:** Rachel Hackett

**Affiliations:** The Company of Biologists, Bidder Building, Station Road, Cambridge CB24 9LF, UK

## Abstract

Disease Models & Mechanisms (DMM) is delighted to announce that the winner of the DMM Outstanding Paper Prize 2021 is Daniel Bronder, for his paper entitled ‘*TP53* loss initiates chromosomal instability in fallopian tube epithelial cells’ ( Bronder et al., 2021). The prize of £1000 is awarded to the first author of the paper that is judged by the journal's editors to be the most outstanding contribution to the journal that year. To be considered for the prize, the first author must be a student or a postdoc of no more than 5 years standing.

## Abstract (precis)

**Summary:** DMM announces Daniel Bronder as the winner of the 2021 DMM Outstanding Paper Prize.

**Figure:**
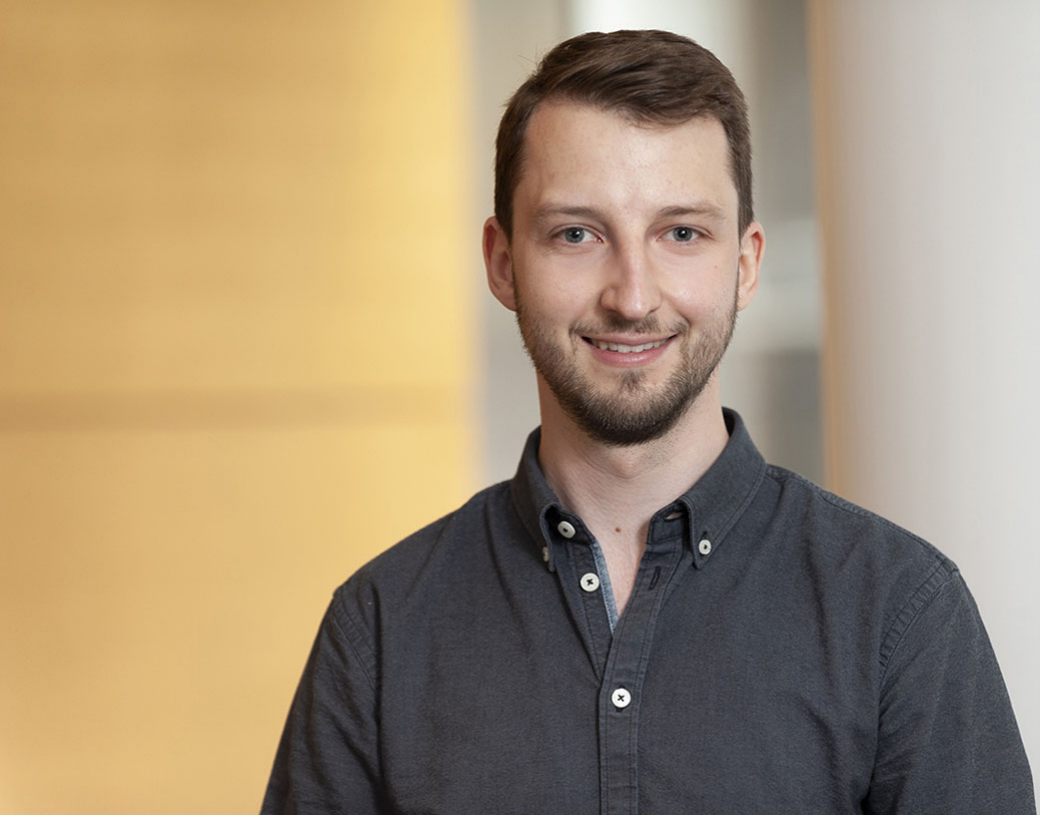
Daniel Bronder

Daniel Bronder's scientific career started with a move from his native Germany to Scotland, where he attended the University of Glasgow and obtained a Bachelor of Science degree in Genetics. During his undergraduate studies, Daniel spent his summers doing internships or student placements in industry and academia. His interest in cancer research was sparked while he interned with Roche Diagnostics in Penzberg, Germany. However, it only came to fruition when he joined Dr Thomas Ried's laboratory at the National Cancer Institute in Bethesda, Maryland, as a summer student.

Following his passion, Daniel joined Prof. Stephen Taylor's laboratory at the Manchester Cancer Research Centre, UK. He joined in 2016 on a Wellcome Trust and National Institutes of Health (NIH) 4-year PhD studentship. This unique program allowed him to initiate a collaboration between Dr Ried's laboratory, which had hosted Daniel for two summers previously, and the laboratory of Prof. Taylor. Daniel's project was designed to allow him to focus on cellular and molecular biology experiments in Manchester, and on molecular cytogenetics and transcriptomics in Bethesda, thus capitalizing on the expertise of the respective host laboratories. This proactive and international PhD program enabled him to experience two different research cultures at an early stage of his career.

During his time as a Wellcome Trust and NIH PhD student, Daniel was actively involved in student government. First, he served as the representative of the Wellcome Trust and NIH student cohort. Later, he chaired the student leadership board of the Oxford and Cambridge Scholars Program at the NIH, which acts as umbrella for Wellcome Trust/NIH PhD students. During his tenure, the student body, much like trainees around the world, faced significant challenges – namely the COVID-19 pandemic. Together with the administrative and academic leadership, the International Biomedical Research Alliance and with buy-in from the student body, social and scholarly events were organized by the student leadership board to maintain an active community. Some of the events initiated in that time have remained or have been adapted as restrictions eased in the US or the UK, and students returned to lab work.

Nonetheless, Daniel was an active student in the lab, where his work focused on chromosomal instability in high-grade serous ovarian cancer. Just before a cell divides, the DNA, which is organized into chromosomes, must be duplicated first and then divided equally between two daughter cells. In cancer cells, this otherwise robust process goes awry, which leads to constant errors in cell division and an imbalance of chromosome number between the daughter cells. For his doctoral thesis work, Daniel investigated whether changes in the DNA sequence of specific genes (i.e. gene mutations) would cause errors in cell division and consequential changes in chromosome number or structure in daughter cells. Ultimately, he could show that changing the DNA sequence of two genes strongly associated with cancer (i.e. *TP53* and *BRCA1*) resulted in increased errors during cell division and chromosome number abnormalities.

Shortly after Daniel submitted his doctoral thesis for examination and the prize-winning manuscript for publication at Disease Models and Mechanisms (Bronder et al., 2021), he moved from Bethesda to New York to take up a position as postdoctoral fellow in Dr Samuel Bakhoum's laboratory at Memorial Sloan Kettering Cancer Center. There, he continues to follow his passion for chromosomal instability in cancer in the hope of exploiting it for patients' benefit.

**Box 1. DMM Prize 2021 shortlist**

**Winner:**

***TP53* loss initiates chromosomal instability in fallopian tube epithelial cells.**

Daniel Bronder, Anthony Tighe, Darawalee Wangsa, Dali Zong, Thomas J. Meyer, René Wardenaar, Paul Minshall, Daniela Hirsch, Kerstin Heselmeyer-Haddad, Louisa Nelson, Diana Spierings, Joanne C. McGrail, Maggie Cam, André Nussenzweig, Floris Foijer, Thomas Ried, Stephen S. Taylor

Disease Models & Mechanisms (2021) 14, dmm049001 doi:10.1242/dmm.049001

**Also shortlisted by our Editor team:**

**A mechanism linking perinatal 2,3,7,8 tetrachlorodibenzo-*p*-dioxin exposure to lower urinary tract dysfunction in adulthood.**

Anne E. Turco, Steven R. Oakes, Kimberly P. Keil Stietz, Cheryl L. Dunham, Diya B. Joseph, Thrishna S. Chathurvedula, Nicholas M. Girardi, Andrew J. Schneider, Joseph Gawdzik, Celeste M. Sheftel, Peiqing Wang, Zunyi Wang, Dale E. Bjorling, William A. Ricke, Weiping Tang, Laura L. Hernandez, Janet R. Keast, Adrian D. Bonev, Matthew D. Grimes, Douglas W. Strand, Nathan R. Tykocki, Robyn L. Tanguay, Richard E. Peterson, Chad M. Vezina

Disease Models & Mechanisms (2021) 14, dmm049068 doi:10.1242/dmm.049068

**An anti-tuberculosis compound screen using a zebrafish infection model identifies an aspartyl-tRNA synthetase inhibitor.**

Eva Habjan, Vien Q. T. Ho, James Gallant, Gunny van Stempvoort, Kin Ki Jim, Coen Kuijl, Daan P. Geerke, Wilbert Bitter, Alexander Speer

Disease Models & Mechanisms (2021) 14, dmm049145 doi:10.1242/dmm.049145

**ELAC2/RNaseZ-linked cardiac hypertrophy in *Drosophila melanogaster***

Ekaterina Migunova, Joanna Theophilopoulos, Marisa Mercadante, Jing Men, Chao Zhou, Edward B. Dubrovsky

Disease Models & Mechanisms (2021) 14, dmm048931 doi:10.1242/dmm.048931

**Heterogeneity in clone dynamics within and adjacent to intestinal tumours identified by Dre-mediated lineage tracing.**

Ann-Sofie Thorsen, Doran Khamis, Richard Kemp, Mathilde Colombé, Filipe C. Lourenço, Edward Morrissey, Douglas Winton

Disease Models & Mechanisms (2021) 14, dmm046706 doi:10.1242/dmm.046706

**Modulation of serotonin in the gut-liver neural axis ameliorates the fatty and fibrotic changes in non-alcoholic fatty liver.**

Masayoshi Ko, Kenya Kamimura, Takashi Owaki, Takuro Nagoya, Norihiro Sakai, Itsuo Nagayama, Yusuke Niwa, Osamu Shibata, Chiyumi Oda, Shinichi Morita, Atsushi Kimura, Ryosuke Inoue, Toru Setsu, Akira Sakamaki, Takeshi Yokoo, Shuji Terai

Disease Models & Mechanisms (2021) 14, dmm048922 doi:10.1242/dmm.048922

**Interpreting the pathogenicity of Joubert syndrome missense variants in *Caenorhabditis elegans*.**

Karen I. Lange, Sofia Tsiropoulou, Katarzyna Kucharska, Oliver E. Blacque

Disease Models & Mechanisms (2021) 14, dmm046631 doi:10.1242/dmm.046631

**Transient, flexible gene editing in zebrafish neutrophils and macrophages for determination of cell-autonomous functions.**

Abdulsalam I. Isiaku, Zuobing Zhang, Vahid Pazhakh, Harriet R. Manley, Ella R. Thompson, Lucy C. Fox, Satwica Yerneni, Piers Blombery, Graham J. Lieschke

Disease Models & Mechanisms (2021) 14, dmm047431 doi:10.1242/dmm.047431
